# Prevalence and characteristics of *Listeria monocytogenes* in ready-to-eat chilled pot skewer products

**DOI:** 10.3389/fmicb.2025.1681344

**Published:** 2025-10-06

**Authors:** Yuzhu Liu, Penghang Zhang, Di Wang, Bowenxin Wang, Yi Zhang, Xiaoyuan Zhang

**Affiliations:** Beijing Key Laboratory of Diagnostic and Traceability Technologies for Food Poisoning, Institute for Nutrition and Food Hygiene, Beijing Center for Disease Prevention and Control (CDC), Beijing, China

**Keywords:** foodborne, *Listeria monocytogenes*, whole-genome sequencing, monitoring, ready-to-eat (RTE)

## Abstract

*Listeria monocytogenes* contamination in ready-to-eat chilled pot skewer products represent a significant public health concern in China. This study assessed the prevalence, molecular characteristics, and potential transmission risks of *L. monocytogenes* in 340 commercially available chilled pot skewer samples collected in 2019 and 2021. Bacterial isolation revealed an overall contamination rate of 20.6% (70/340), with no significant differences among meat-based (15.5%), vegetarian (19.5%), or mixed meat-vegetable (26.5%) products. Serogroups 1/2b, 3b, 7 (47.4%) and 1/2a, 3 (35.5%) predominated. Multilocus sequence typing (MLST) identified high-risk clones ST87 (27.6%) and ST121 (22.4%)—both associated with listeriosis outbreaks—along with rare lineages such as ST378 (1.3%). Core genome MLST (cgMLST) analysis of 76 isolates revealed extensive genetic diversity (59 cgMLST types), suggesting multiple contamination sources within production facilities. However, persistence of specific strains (≤ 2 allelic differences) across time points (2019–2021) was observed in certain manufacturers. Comparative genomics with clinical isolates from Beijing (2013–2022) revealed shared sequence types (e.g., ST87/CC87). These findings underscore the urgent need for enhanced surveillance in food processing environments. Future investigations should adopt a One Health framework to trace contamination routes and evaluate intervention control measures for this emerging food safety challenge.

## Introduction

*Listeria monocytogenes* is a Gram-positive, facultative intracellular pathogen that remains a significant threat to global public health as one of the most virulent foodborne pathogens (Radoshevich and Cossart, 2018). This bacterium is particularly concerning due to its ability to withstand a wide array environmental stresses. It can survive and proliferate across a broad temperature range (−0.4 °C to 45 °C), pH values ranging from 4.6 to 9.5, and low water activity (aW < 0.90), and it tolerates salt concentrations up to 20% (Osek and Wieczorek, 2023). Human listeriosis is characterized primarily by severe invasive infections such as septicemia, meningitis, and encephalitis, with case fatality rates reaching 20–30% among high-risk populations, including pregnant women, neonates, the elderly, and immunocompromised individuals (McCollum et al., 2013; Wang et al., 2013). Despite rigorous food safety standards implemented worldwide, *L. monocytogenes* outbreaks continue to occur sporadically in both developed and developing countries (Halbedel et al., 2023; Kleta et al., 2017; McLauchlin et al., 2020; Smith et al., 2019).

Addressing the threat of *L. monocytogenes* requires a One Health approach, which recognizes that the health of humans, animals, and the environment are inextricably linked. Food is the principal source of *L. monocytogenes* exposure in humans (Wiktorczyk-Kapischke et al., 2023). The primary route of human infection is the consumption of contaminated ready-to-eat (RTE) foods that support *Listeria* growth without requiring further cooking. The safety of RTE foods is a persistent challenge, as their production often involves extensive handling and they are frequently exposed to contamination in processing environments, where pathogens can form resilient biofilms (Colagiorgi et al., 2017; Galié et al., 2018). In recent years, epidemiological investigations have linked listeriosis outbreaks to a wide range of RTE products, including deli meats (Smith et al., 2019; Niu et al., 2024), soft cheeses (Sévellec et al., 2020), smoked seafood (Halbedel et al., 2023), prepackaged salads (Harter et al., 2017), and ice cream (Conrad et al., 2023; Zhang et al., 2025). However, limited attention has been given to another rapidly expanding category: Asian-style RTE street foods adapted for large-scale commercial production. One example is Chinese-style chilled pot skewer—a dish consisting of skewered meats, seafoods, vegetables, and tofu, served fresh or pre-packaged with spicy broth. These products raise particular concern due to their complex multi-ingredient composition, extended handling during preparation, susceptibility to temperature abuse, and lack of a terminal heat-treatment (kill step) prior to consumption.

Whole genome sequencing (WGS) has revolutionized the study of *L. monocytogenes*, offering unprecedented resolution for outbreak investigation, virulence profiling, and global surveillance (Moura et al., 2016). By enabling high-precision source tracking, WGS links clinical isolates to contaminated food products and processing environments, thereby uncovering persistent strains and cross-contamination within production facilities (Halbedel et al., 2023; Kleta et al., 2017; McCollum et al., 2013; Niu et al., 2024; Pérez-Trallero et al., 2014; Schjørring et al., 2017; Smith et al., 2019). It also enhances the analysis of virulence and persistence by identifying key genomic islands (e.g., LIPI1 to LIPI4 and SSI-1/2) associated with biofilm formation, stress tolerance, and pathogenic potential (Ji et al., 2023; Maury et al., 2019; Zhang X. et al., 2021). Furthermore, WGS has transformed public health surveillance systems by replacing traditional pulsed-field gel electrophoresis (PFGE) with rapid and discriminatory single-nucleotide polymorphism (SNP)-based clustering methods. Its integration into global genomic databases facilitates real-time data sharing, thereby strengthening cross-border outbreak response capabilities. Additionally, WGS supports microbial risk assessment by distinguishing high- and low-risk clones based on genetic markers linked to virulence and antimicrobial resistance (Maury et al., 2019). Crucially, WGS is an indispensable tool for comprehensively characterizing antimicrobial resistance (AMR) genes in foodborne isolates, predicting resistant phenotypes, and monitoring the emergence and spread of resistant clones across the food chain (Chen et al., 2020; Kurpas et al., 2020; Moura et al., 2024). As a cornerstone of modern food safety and public health, WGS continues to drive advancements in the detection, prevention, and control of *L. monocytogenes*.

Despite the widespread application of WGS, no WGS-based study has examined *L. monocytogenes* contamination in chilled pot skewer samples, a product that combines multiple high-risk ingredients and exhibits RTE consumption patterns similar to those of deli meats. In this study, we aimed to (1) determine the prevalence of *L. monocytogenes* across chilled pot skewer samples using culture-based methods; (2) characterize the dominant sequence types through serogrouping, multilococus sequence typing (MLST), and core genome MLST (cgMLST) analysis; and (3) compare food-derived isolates with clinical isolates from Beijing to inform risk prioritization. This work addresses critical knowledge gaps in an underregulated food sector and highlights the utility of WGS in proactive food safety risk management.

## Materials

### Samples

A total of 340 chilled pot skewer samples were analyzed. Chilled pot skewers is a traditional Chinese snack consisting of various vegetables and meats skewered with bamboo sticks, cooked in a specially formulated broth, cooled and served in a seasoned cold soup. The product is typically categorized into three types: meat-only skewers, vegetarian skewers, and mixed meat-and-vegetable skewers. In this study, the sample set included 110 meat skewers, 128 vegetarian skewers, and 102 mixed skewers. The 340 samples were collected from 57 different manufacturers, labeled A through ZZ (Supplementary Table 1).

### Isolation of *L. monocytogenes*

Isolation of *L. monocytogenes* was performed according to the GB 4789.30-2016 standard (National Food Safety Standard Food Microbiological Examination Examination of Listeria monocytogenes, 2016). Each 25-g food sample was mixed with 225 mL of LB1 medium (Beijing JUNLIKANG Technology Development CO., LTD, Beijing, China) for primary selective enrichment and homogenized using a stomacher. The mixture was incubated at 30 °C for 18 h. For the second enrichment, 0.1 mL of the primary culture was transferred to 10 mL of LB2 medium (Beijing JUNLIKANG Technology Development CO., LTD, Beijing, China) and incubated at 30 °C for 24 h. A loopful of the LB2 culture was streaked onto chromogenic medium for *L. monocytogenes* (CHROMagar, Paris, France) and incubated at 37 °C for 24 h. Five presumptive colonies from each plate were subcultured onto blood agar (Thermo Fisher Scientific Inc., Waltham, Massachusetts, USA), a non-selective medium, and incubated at 37 °C for 24–48 h. Colonies grown on blood agar were confirmed as *L. monocytogenes* through Gram staining, standard biochemical tests, and matrix-assisted laser desorption/ionization time-of-flight mass spectrometry (MALDI-TOF MS) (Bruker Corporation, Billerica, Massachusetts, USA).

### Genomic DNA extraction and WGS

*Listeria monocytogenes* isolates were routinely cultured in brain heart infusion broth at 37 °C overnight. Genomic DNA was extracted using a commercial kit (DNeasy UltraClean Microbial Kit, Qiagen, Germany) according to the manufacturer’s instructions. WGS was performed on an Illumina NovaSeq apparatus (Illumina, San Diego, CA, USA) using two paired-end libraries with average insertion sizes of 350 bp and 2,000 bp. Raw sequencing reads were quality filtered by removing reads containing more than five ambiguous bases, reads with more than 20 bases below Q20, adapter-contaminated sequences, and duplicate reads. After filtering, high-quality paired-end data were retained, yielding 100 × coverage for each library.

### Serotype, sequence type, and core genome multilocus sequence typing of *L. monocytogenes*

Raw WGS data were imported into BioNumerics version 7.6 (Applied Maths, Oost-Vlaanderen, Belgium) and uploaded to the National Molecular Tracing Network for Foodborne Diseases Surveillance (TraNet) calculation engine at Aliyun (Alibaba Group, Hangzhou, China) for *de novo* genome assembly. Serotyping of *L. monocytogenes* strains was performed using LisSero^1^, while sequence type (ST) assignment and clonal complex (CC) were conducted using the mlst tool^2^.

### Virulence and resistance gene profiles

Virulence-associated genes were identified by analyzing isolates against the Virulence Factor Database (VFDB) on August 15th, 2023, using a minimum identity threshold of 75% and a coverage threshold of 60%. Antimicrobial resistance genes were analyzed using ResFinder 3.0 (Center for Genomic Epidemiology) with parameters set at a minimum of 90% identity and 60% coverage. Genes related to pathogenicity islands, internalins, adherence, invasion, stress, intracellular growth, immunomodulator, peptidase function, immune evasion, and bile resistance were investigated.

### Nucleotide sequence accession number

Data is deposited in National Microbiology Data Center (NMDC) with accession numbers NMDC10019942^3^.

### Antimicrobial susceptibility testing

Antimicrobial susceptibility testing of *L. monocytogenes* isolates was performed using the broth dilution method. We measured the minimum inhibitory concentrations (MICs) of ampicillin (AMP), penicillin (PEN), tetracycline (TET), meropenem (MRP), trimethoprim-sulfamethoxazole (SXT), erythromycin (ERY), vancomycin (V AN), and ciprofloxacin (CIP; Xingbai, Shanghai, China). The MICs of AMP, PEN, SXT, and MRP were interpreted according to the Clinical and Laboratory Standard Institute (CLSI) guidelines, while the MIC of ERY was interpreted using the European Committee on Antimicrobial Susceptibility Testing (EUCAST) guidelines. Considering that no resistance breakpoints have been established for *L. monocytogenes* for TET, VAN, or CIP, the MICs for these three antimicrobials were interpreted based on criteria for *Staphylococcus* spp. ATCC29213, which was used as the reference strain.

### Statistical analysis methods

The chi-square test (χ^2^ test) was used to analyze differences in *L. monocytogenes* positive detection rates, serogroup distribution, and ST distribution among different sample types, and the difference in isolation rates between 2019 and 2021.

## Results

### Prevalence of *L. monocytogenes*

Using culture-based methods, *L. monocytogenes* was detected in 20.6% (70/340) of the chilled pot skewer samples. The positive detection rate of *L. monocytogenes* was 14% (14/100) in 2019 and 23.3% (56/240) in 2021, with no statistically significant difference (χ^2^ = 3.761, *p* = 0.052). Specifically, the prevalence was 15.5% (17/110) in meat skewer samples, 19.5% (17/128) in vegetarian skewer samples, and 26.5% (27/102) in mixed meat-and-vegetable skewer samples. No statistically significant differences in prevalence, serogroup distribution and ST distribution were observed among the three categories (Supplementary Table 3). All *L. monocytogenes* isolates originated from samples collected from 11 manufacturers, while no isolates were detected from the remaining 46 manufacturers (Table 1). There were 5 manufacturers (R, E, H, O, Z) from which samples were collected for *L. monocytogenes* detection in both 2019 and 2021. Among these, *L. monocytogenes* isolates were detected in 2 manufacturers (R, E) in both 2019 and 2021. No isolates was detected in the other 3 manufacturers (H, O, Z)in either 2019 or 2021. In six of the 70 positive samples, two isolates were obtained per sample, resulting in a total of 76 isolates included in this study. The information of these isolates were shown in Supplementary Table 2.

**Table 1:** *Listeria monocytogenes* isolates recovered from samples collected from 11 manufacturers.

Manufacturer (Isolation time)	Number of Samples	Number of positive Samples (percentage)	Number of Isolates	Serotype (Number)	ST (Number)	Number of CgMLST
SS (2021)	8	8 (100%)	10	1/2a,3a (2), 1/2b,3b,7 (8)	37 (1), 87 (5), 121 (1), 716 (1), 717 (1)	7
D (2019)	3	2 (66.67%)	2	1/2a,3a (2)	121 (1), 8 (1)	2
E (2019)	10	8 (11.59%)	8	1/2a,3a (1), 1/2b,3b,7 (7),	3 (2), 5 (4), 87 (1), 121 (1)	6
E (2021)	59	27 (39.13%)	31	1/2a,3a (14), 1/2b,3b,7 (15), 1/2c,3c (2)	3 (3), 5 (3), 9 (2), 87 (7), 121 (13), 378 (1), 3,297 (2)	27
AB (2021)	8	4 (50%)	4	1/2a,3a (1), 1/2b,3b,7 (3)	155 (1), 87 (3)	4
A*F* (2021)	2	1 (50%)	1	1/2c,3c (1),	9 (1)	1
BB (2019)	2	1 (50%)	1	1/2a,3a (1)	8 (1)	1
TT (2021)	16	4 (25%)	4	1/2a,3a (2), 1/2b,3b,7 (1), 1/2c,3c (1)	5 (1), 8 (2), 9 (1)	4
PP (2021)	8	2 (25%)	2	1/2a,3a (1), 1/2b,3b,7 (1)	37 (1), 87 (1)	2
MM (2019)	4	1 (25%)	1	1/2a,3a (1)	121 (1)	1
F (2021)	38	6 (15.79%)	6	1/2c,3c (6)	1,113 (6)	2
R (2019)	8	2 (6.25%)	2	1/2a,3a (2),	101 (2)	2
R (2021)	24	4 (12.5%)	4	1/2b,3b,7 (1), 1/2c,3c (3)	87 (1), 1,113 (3)	3

### Serogroup and MLST analysis

Almost half of the isolates belonged to serogroup 1/2b, 3b, 7 (*n* = 36, 47.4%), followed by serogroup 1/2a, 3a (*n* = 27, 35.5%) and serogroup 1/2c, 3c (*n* = 13, 17.1%). No isolates belonging to serogroup 4b, 4d, 4e were detected. Seventy-six isolates were distributed across 10 CCs and 14 STs. The most common CCs were CC87 (*n* = 23, 30.3%), comprising four STs (ST87 *n* = 19; ST3297 *n* = 2; ST716 *n* = 1; ST717 *n* = 1) and CC121/ST121 (*n* = 17, 22.4%), followed by CC9 (*n* = 13, 17.1%) comprising two STs (ST1113 *n* = 9; ST9 *n* = 4), CC5/ST5 (*n* = 8, 10.5%), CC3/ST3 (*n* = 5, 6.6%), CC8/ST8 (*n* = 4, 5.3%), CC101/ST101 (*n* = 2, 5.3%), CC37/ST37 (*n* = 2, 5.3%), CC155/ST155 (*n* = 1, 1.3%), and CC19/ST378 (*n* = 1, 1.3%). Not all isolates from the same manufacturer shared the same serogroup or ST. Additionally, isolates from different manufacturers exhibited distinct STs. Some of the ST of strains isolated from manufacturer E in 2019 and 2021 were the same, while the ST of strains isolated from manufacturer R in different years were all different (Table 1).

### Cluster detection using cgMLST analysis

**Figure 1:**
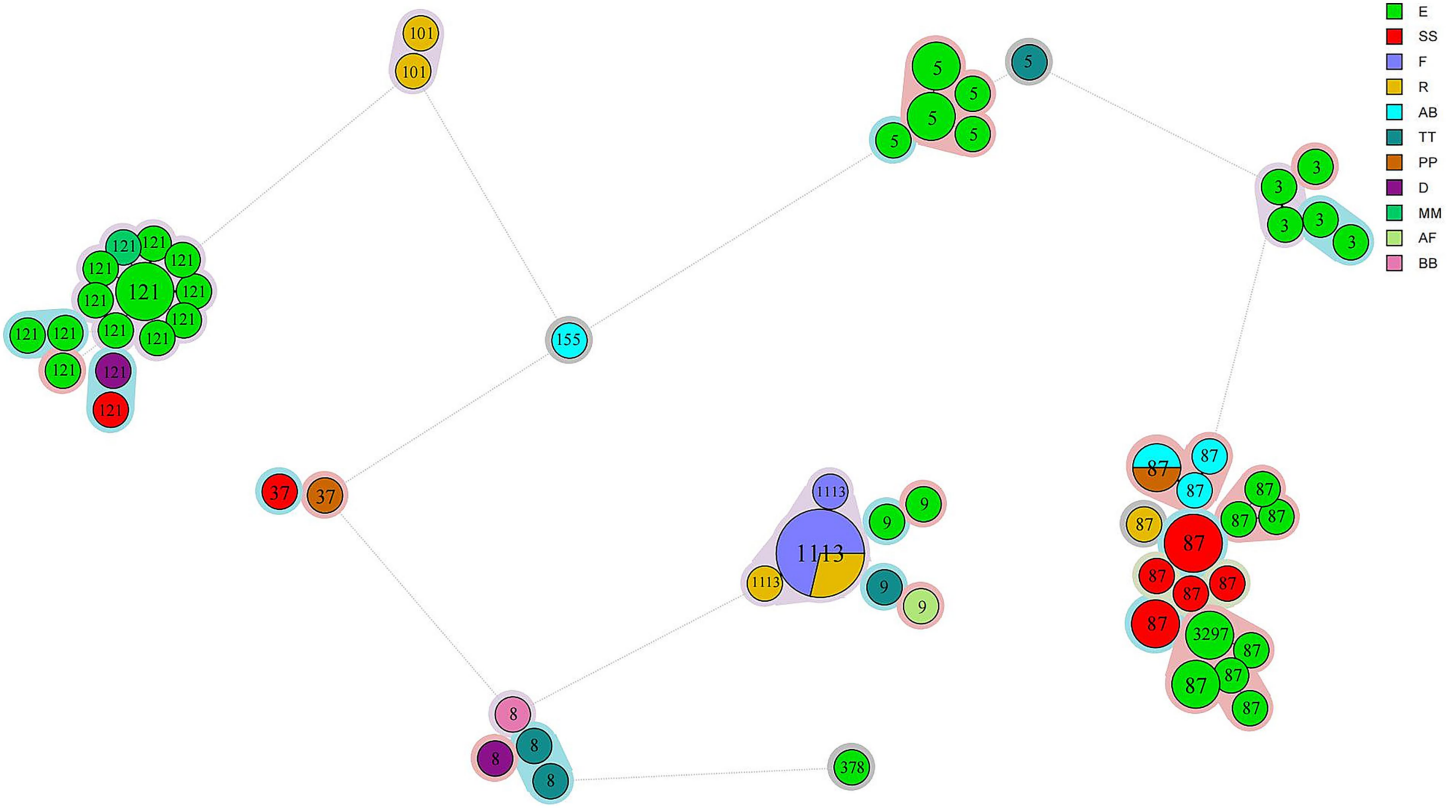
Phylogeny of 76 *L. monocytogenes* isolates obtained from chilled pot skewer products. A minimum spanning tree based on cgMLST analysis is shown. Each circle represents one cgMLST type. Circle size is proportional to the number of isolates, and colors indicate manufacturer source. STs are labeled in circles. Color shading around clusters denotes groups with ≤10 allelic differences.

cgMLST analysis separated all 76 isolates into 59 cgMLST types (Figure 1). Isolates from the same manufacturer did not always cluster together. For example, 39 isolates from manufacturer E were distributed across 33 cgMLST types, with more than 200 allelic differences observed between some types. These 33 cgMLST types were grouped into five clusters. Ten isolates from manufacturer SS were assigned to seven cgMLST types; among them, eight isolates formed a single cluster with a maximum of 16 allelic difference. Six isolates from manufacturer F belonged to a single cluster with a maximum of two allelic differences. Six isolates from manufacturer R were assigned to three clusters. In total, 61 isolates were grouped into 15 cgMLST complexes, each defined with ≤ 10 allelic differences between pairs of neighboring isolates (Table 2). These 15 complexes contained between two and twelve isolates and were derived from one to two manufactures and one to two STs (Table 2). Eleven complexes included isolates from a single manufacturer, while the remaining four included isolates from two manufacturers. Complexes C3, C13, and C15, originating from manufacturer E, contained strains collected during two distinct sampling periods (2019 and 2021), corresponding to ST5, ST87, and ST121, respectively.

**Table 2:** Fifteen complexes with ≤10 allelic differences between pairs of neighboring isolates.

Complexes	Isolates number	Isolates	Maximum number of cgMLST alleles	ST	Serogroup (Number)	Manufacturer (Number)
C1	9	698,625,685,694,623,699,581,695,624	2	1,113	1/2c,3c(9)	F(6), R(3)
C2	2	673,639	7	8	1/2a,3a(2)	TT(2)
C3	12	575,571,574,671,446,583,494,697,576,668,696,666	10	121	1/2a,3a(12)	E(11), MM(1)
C4	2	465,682	9	121	1/2a,3a(2)	D(1), SS(1)
C5	2	577,676	4	121	1/2a,3a(2)	E(2)
C6	2	439,440	4	101	1/2a,3a(2)	R(2)
C7	2	443,444	1	3	1/2b,3b,7 (2)	E(2)
C8	2	675,677	6	3	1/2b,3b,7 (2)	E(2)
C9	2	684,716	0	87	1/2b,3b,7 (1), 3b(1)	SS(2)
C10	3	683,681,663	0	87	1/2b,3b,7 (3)	SS(3)
C11	3	662,717,579	7	87	1/2b,3b,7 (2), 3b(1)	SS(3)
C12	7	665,670,611,612,680,678,582	10	87,3,297	1/2b,3b,7 (7)	E(7)
C13	3	679,445,669	3	87	1/2b,3b,7 (3)	E(3)
C14	4	593,596,712,713	2	87	1/2b,3b,7 (4)	PP(1), AB(3)
C15	6	473,447,442,441,664,700	8	5	1/2b,3b,7 (6)	E(6)

### Comparison of chilled pot skewer isolates with human clinical isolates from Beijing (2013–2022)

A comparative genomic analysis was conducted between the 76 *L. monocytogenes* isolates obtained from chilled pot skewers products in this study and 275 clinical isolates from listeriosis patients in Beijing collected between 2013 and 2022. WGS revealed that 18 isolates shared ≤ 10 allelic differences in cgMLST profiles with clinical strains, suggesting potential epidemiological linkage. The 18 isolates belong to three STs: ST87 (*n* = 15), originating from four manufacturers (SS: 8 isolates; AB: 3; E: 3; PP: 1); ST8 (*n* = 2), each from manufacturers TT and BB; and ST378 (*n* = 1), from manufacturer E. All strains were classified into serogroup 1/2b, 3b, 7. We conducted a retrospective epidemiological investigation on these relevant patients and analyzed their epidemiological data, and no association was established at the epidemiological level.

### Virulence and stress-adaptation genes in *L. monocytogenes* isolates

**Figure 2:**
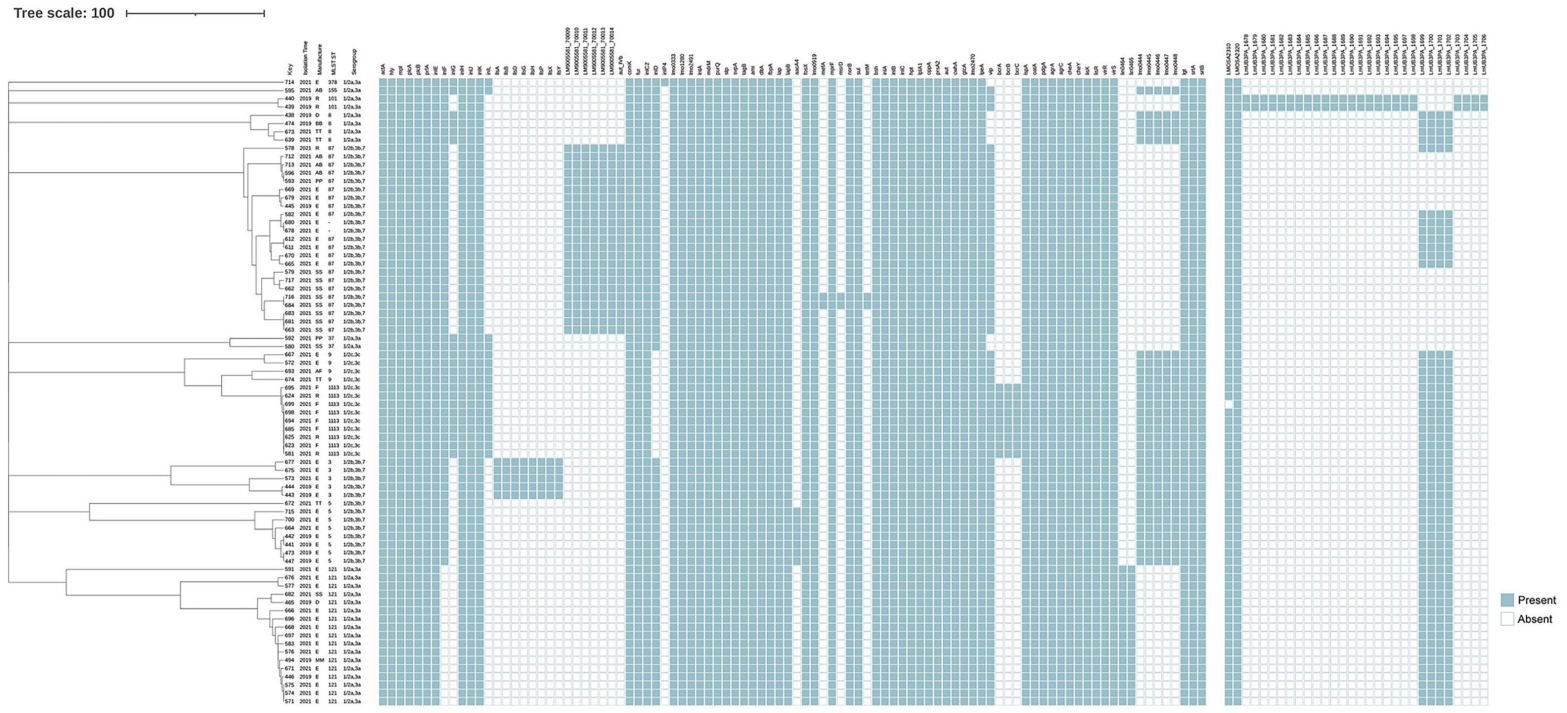
Virulence and antibiotic resistance profiles mapped onto the phylogeny of 76 *L. monocytogenes* isolates. The dendrogram on the left is annotated with isolate ID, year of isolation, manufacturers, ST, and serogroup. A binary presence/absence matrix displays genes detected in each isolate, characterized as follows: pathogenicity islands LIPI-1 (*actA, hly, mpl, plcA, plcB, prfA*), LIPI-2 (*inlE, inlF, inlG, inlH, inlJ, inlK, inlL*), LIPI-3 (*llsA, llsG, llsH, llsX, llsB, llsY, llsD, llsP*), and LIPI-4 (LM9005581_70009 to LM9005581_70014); stress survival islands SSI-1 (lmo0444 to lmo0448) and SSI-2 (lin0464, lin0465); antibiotic resistance genes (*aacA4, fosX*, lmo0919, *mefA, mprF, msrD, norB, sul, tetM*); disinfectant resistance genes (*bcrABC*); *Listeria* genomic islands LGI-2 (LMOSA2310, LMOSA2320) and LGI-3 (LmUB3PA_1678 to 1,693); genes involved in adherence (*ami, fbpA, dltA, lap, lapB*); invasion-related genes (*aut, cwhA, lpeA, vip, gtcA,* lmo2470); intracellular growth genes (*hpt, lplA1, oppA, prsA2*); surface protein-anchoring genes (*lgt, srtA, srtB*); internalin genes (*inlABC*); regulatory genes (*agrA, agrC, cheA, cheY, lisK, lisR, virR, virS*); and other functional genes such as peptidase (lspA), peptidoglycan modification (oatA, pdgA), bile resistance (*bsh*), and other virulence genes (*aut_IVb, comK, fur, inlC2, inlD,* lmo0333, lmo1280, lmo2491*, lntA, mdrM, purQ, stp, svpA, tagB*).

All 76 *L. monocytogenes* isolates were screened for stress adaptation-related genetic elements, including stress survival islet 1 (SSI-1), stress survival islet 2 (SSI-2), three *Listeria* genomic islands (LGI1, LGI2, and LGI3), and two benzalkonium chloride resistance determinants: the *bcrABC* gene cassette and the *ermC* gene. SSI-1 was present in 31 isolates (40.8%), specifically in nine ST1113 isolates, four ST9 isolates, four ST8 isolates, and one ST9 isolate. SSI-2 was found exclusively in all 17 ST121 isolates (Figure 2). LGI-1 was absent in all isolates. In the case of LGI-2, two of its 34 constituent genes were consistently present in all 76 isolates, except for isolate 699, which contained only one gene (*LMSA2320*). A variant form of LGI-3 was identified in two ST101 isolates. This variant lacked four genes (*LmUB3PA_1699* to *LmUB3PA_1702*) compared to the LGI-3 initially described in ST101 strain A37-02-LmUB3PA by Palma et al. (2020). The missing genes encode a Tn3 family transposase, a *hin* recombinase, and cadmium resistance genes (*cadC* and *cadA1*). The *bcrABC* gene cassette was detected exclusively in all ST121 isolates, whereas the *ermC* gene was absent in all isolates.

All isolates were screened for the presence of four *L. monocytogenes*-specific pathogenicity islands (LIPIs): LIPI-1, LIPI-2, LIPI-3, and LIPI-4. LIPI-1 was highly conserved and present in all 76 isolates. Among the LIPI-2 internalin genes, *inlE*, *inlH*, *inlJ*, and *inlK* were detected across all isolates, whereas *inlF*, *inlG*, and *inlL* were found only in a subset. Notably, *inlF* was absent in all ST121 isolates. *InlG* and *inlL* were present in isolates belonging to ST378, ST155, ST37, ST9, and ST1113, while ST8 isolates carried *inlG* but lacked *inlL*. LIPI-3 was exclusively identified in ST3 isolates. LIPI-4 was identified in all ST87 isolates and in ST3297, which belongs to the clonal complex CC87. The *inlA* gene, which encodes full-length Internalin A (InlA), was intact in all 76 *L. monocytogenes* isolates. However, point mutations in *inlA* were observed in eight isolates, specifically in ST3 (3/5), ST121 (2/17), ST155 (1/1), ST87 (1/21), and ST5 (1/8). Isolates of the same ST exhibited identical *inlA* point mutation patterns.

### Antibiotic resistance genes in *L. monocytogenes* isolates

All 76 isolates were susceptible to CIP, VAN, MRP, SXT, and AMP. Two isolates (684 and 716) were resistant to both TET and ERY. Resistance phenotypes were consistent with genotypic profiles. All isolates contained five intrinsic antibiotic resistance genes, including *fosX* (resistance to fosfomycin), lmo0919 (lincosamides), *norB* (quinolones), *mprF* (cationic antimicrobial peptides), and *sul* (sulfonamides). Additionally, four acquired antibiotic resistance genes were identified in a subset of isolates. The aminoglycosides resistance gene *aacA4* was detected in seven ST5 isolates, located with a prophage inserted between the *smc* gene and a hypothetical protein gene. The *tetM*, *mefA*, and *msrD* genes were found in only two ST87 isolates (684 and 716); *tetM* was located within a prophage, while *mefA* and *msrD* were integrated into the chromosome.

## Discussion

This study revealed that the isolation rate of *L. monocytogenes* in chilled pot skewer products increased from 14.0% in 2019 to 23.3% in 2021 with no statistically significant difference, with an overall positivity of 20.3% (69/340). As an RTE food, Chilled pot skewers pose multiple risk points for *L. monocytogenes* contamination. For example, (1) raw material handling: if ingredients such as meat or seafood are not thoroughly cooked, *L. monocytogenes* may be introduced; (2) post-cooking contamination: skewers may become recontaminated during improper storage or handling after cooking; and (3) broth exposure: if the broth is left exposed to the environment for extended periods, it may serve as a reservoir for *L. monocytogenes.* While there have been no prior reports specifically addressing *L. monocytogenes* contamination in chilled pot skewer products, its presence in RTE foods is well-documented and has been associated with large-scale outbreaks. A meta-analysis conducted in China reported a prevalence of 8.5% in raw meats and 3.2% in RTE meats (Liu et al., 2020), both lower than the rate observed in our study. Between 2017 and 2018, South Africa experienced the largest listeriosis outbreak, which was linked to RTE processed meats contaminated with *L. monocytogenes*, resulting in 1,060 confirmed cases and 216 deaths (Smith et al., 2019). In the United States, *L. monocytogenes* must be absent in RTE foods at any point during production (U.S Food Safety and Inspection Service, 2003). Similarly, the Chinese national food standard GB29921 stipulates that *L. monocytogenes* must not be detectable in 25 g or 25 mL prepackaged RTE food products (National Food Safety Standards Limits of Pathogenic Bacteria in Prepackaged Food, 2021). All *L. monocytogenes* isolates in this study originated from 11 manufacturers, while no isolates were detected in samples from the remaining 46 manufacturers. This suggests that contamination may be concentrated in specific production facilities.

The dominant serogroups identified in this study were 1/2b, 3b, 7 (47.4%) and 1/2a, 3a (35.5%), with no detection of serogroup 4b, an important serogroup globally associated with severe human listeriosis cases. This serological profile aligns with the distribution patterns observed in Chinese RTE foods (Chen et al., 2020; Ji et al., 2023; Zhang et al., 2020), but differs notably from international findings. For example, in Polish RTE meat products, isolates were classified into four serogroups: IVb (33%, 17/52), IIa (29%, 15/52), IIc (19%, 10/52), and IIb (19%, 10/52) (Maćkiw et al., 2020). Notably, the serogroup distribution in chilled pot skewer products in our study contrasts sharply with that reported for raw meat isolates in China, where serogroups IIa and IIc predominated (Ji et al., 2023; Li et al., 2022). The potential reasons for the differing serogroups of *L. monocytogenes* between chilled pot skewers and raw meat sources may be: Firstly, variations in raw material composition – unlike raw meat, chilled pot skewers are composite foods containing not just meat but also vegetables, soy products and other ingredients, each potentially carrying *L. monocytogenes* strains of different serotypes. Secondly, the selective effect of processing and storage – chilled pot skewers undergo cooking followed by sauce soaking, a process that may eliminate strains with lower environmental resistance. However, it closely mirrors the serotype distribution of *L. monocytogenes* strains isolated from clinical listeriosis cases in China (Li et al., 2023; Zhang X. et al., 2021). Our previous studies have also indicated that ready-to-eat (RTE) foods, including Chinese-style chilled dishes, are high-risk factors for *L. monocytogenes* infection (Niu et al., 2022).

Our study identified 12 distinct *L. monocytogenes* STs, with ST87, ST121, ST1113, and ST5 representing the predominant lineages. These STs are consistent with previous findings on both clinical and foodborne isolates in China, where ST87, ST121, and ST5 are frequently associated with human listeriosis cases (Li et al., 2023; Zhang X. et al., 2021). Notably, ST87 emerged as the most prevalent lineage across multiple food matrices, including RTE foods, pasteurized milk (Chen et al., 2020; Cheng et al., 2022), aquatic products, and edible mushrooms (Chen et al., 2018a, 2018b), paralleling its clinical prominence in China (Zhang X. et al., 2021). This dual prevalence of ST87 in food and clinical settings underscores its significance in public health. The clinical predominance of ST87 may stem from its unique genetic composition, including the pathogenicity island LIPI-4 and the conserved plasmid pLM1686, which contribute to stress tolerance and enhanced pathogenicity (Wang et al., 2019). Its persistent detection in packed salmon (4-year span) (Chau et al., 2017) and mushroom facilities (Sun et al., 2021) highlights exceptional environmental resilience. ST121 was the third most prevalent ST in food in China (Ji et al., 2023). ST121 is commonly found in foods, particularly in meat and meat products, and in food production plants due to its carriage of SSI2 and robust biofilm formation capacity, which may explain its high prevalence in our study (Harter et al., 2017; Liu et al., 2022; Maury et al., 2019). ST5 has been widely reported in foods and food processing environments (Kurpas et al., 2020; Naditz et al., 2019; Zhang et al., 2021a). Zhang et al. showed the ST5 isolates are capable of forming biofilms, providing a protective environment that enhances bacterial survival and increases the risk of recurrent contamination (Zhang et al., 2020). ST1113, belonging to serogroup 1/2c, 3c, has not yet been identified in clinical strains. However, in our study, ST1113 was the only ST that harbored disinfectant resistance-associated genes, which may explain its relatively high prevalence in chilled pot skewer products.

cgMLST analysis revealed that the 76 *L. monocytogenes* isolates were classified into 59 genotypes, indicating a high level of genetic diversity. Among these, 39 isolates from manufacturer E were distributed across 33 cgMLST types, suggesting long-term, multi-source contamination within the processing environment. This may result from persistent biofilm residues on equipment surfaces or cross-contamination of raw materials. Similar findings have been reported in studies examining *L. monocytogenes* diversity in frozen vegetables and RTE processing plants (Truchado et al., 2022; Zhang et al., 2021a). In contrast, six isolates from manufacturer F were grouped in a single cluster (≤2 allelic differences), indicating a likely point-source contamination event, potentially due to a specific batch of raw materials or equipment contamination. The presence of cgMLST complexes (e.g., C3, C13, and C15) may reflect shared supply chains (e.g., common raw material suppliers or logistics networks) leading to cross-contamination. Notably, ST5, ST87, and ST121 were detected in samples collected from 2019 to 2021, indicating persistent environment contamination sources at manufacturer E. This aligns with the One Health perspective, where food processing plants represent a critical interface where environmental persistence in biofilms directly leads to food contamination, posing a risk to human health. Our findings underscore the need for enhanced environmental monitoring and sanitation protocols in these facilities to break this cycle.

Our study compared *L. monocytogenes* isolates recovered from chilled pot skewer products with clinical isolates from listeriosis patients in Beijing between 2013 and 2022. WGS revealed that 18 isolates exhibited ≤ 10 allelic differences in their cgMLST profiles relative to clinical strains, suggesting a potential common origin or transmission chain. Even though direct epidemiological evidence is lacking, and causality cannot be confirmed, these findings highlight that chilled pot skewer products represent a significant food safety concern. The 18 closely related isolates belonged to three STs: ST87 (*n* = 15), ST8 (*n* = 2), and ST378 (*n* = 1), classified under serogroup 1/2b, 3b, 7. ST87 is rare in countries outside China. In 2014, Perez-Trallero et al. reported two outbreaks in Northern Spain involving ST87, one of which was linked to contaminated foie grass and affected 15 individuals (Pérez-Trallero et al., 2014). In China, although large outbreaks caused by ST87 have not been reported, this ST is frequently associated with sporadic listeriosis cases. For example, a pregnant woman in Zigong was infected after consuming cold mixed cooked food from a farmers’ market (Zhang et al., 2018), and a case in Beijing was traced to contaminated pre-packaged cold-chain RTE food, with genetically identical ST87 strains also isolated from raw materials and the food production environment (Niu et al., 2024). ST8 has been implicated in multiple international outbreaks. From 2015 to 2017, it was responsible for a multinational listeriosis outbreak linked to cold-smoked salmon in Denmark and France (Schjørring et al., 2017). Outbreaks involving ST8 have been reported in Germany (Fischer et al., 2021), and an case in Beijing was linked to the consumption of contaminated ice cream (Zhang et al., 2025). Although ST378 has not been associated with listeriosis outbreaks in China, it has been isolated from clinical cases within the country (Zhang X. et al., 2021), as well as RTE meat processing plants (Zhang et al., 2021b). Additionally, ST378 was detected in animal-derived food items confiscated from passengers arriving at the Bilbao International Airport in Spain from non-European countries (Rodríguez-Lázaro et al., 2015).

The overall acquired antimicrobial resistance profile of the 76 *L. monocytogenes* isolates in this study was relatively mild, which was consistent with the global consensus that *L. monocytogenes* exhibits lower resistance rates compared to other common foodborne pathogens such as *Salmonella* spp. and *Staphylococcus aureus* (Li et al., 2023; Moura et al., 2024). In this study, all 76 isolates were susceptible to CIP, VAN, MRP, SXT, and AMP, while two isolates were resistant to both TET and ERY. Tetracycline resistance is a common phenotype in *L. monocytogenes* isolates from humans and food, with the *tetM* gene being the most common resistance genotype (Escolar et al., 2017; Moura et al., 2024; Olaimat et al., 2018). The two strains in this study also carried the *tetM* gene. Although no acquired multidrug resistance (MDR) isolate was detected in our study, national food monitoring data in China revealed an increase in the acquired MDR detection rate of *L. monocytogenes* from 0.38 to 0.42% (Li et al., 2025; Yan et al., 2019). Moreover, it was found that except for CC9 and CC87/ST87, CC8/ST8 and CC155/ST705 showed high virulence and MDR patterns (Li et al., 2025). In this study, we detected aminoglycoside resistance gene *aacA4* in seven ST5 isolates, *tetM, mefA*, and *msrD* genes in two ST87 isolates. Although we did not perform phenotypic testing for aminoglycoside resistance in this study, we could not determine the relationship between phenotype and genotype. However, a study in France found that isolates harboring *aacA4* or *aphA* did not exhibit resistance phenotypes to aminoglycosides (Moura et al., 2024). Additionally, ST87 and ST5 are among the top three ST types in clinical isolates in China, especially those affecting neonatal cases and central nervous system infections (Li et al., 2023; Zhang X. et al., 2021). The *tetM* and *aacA4* genes were located within a prophage, as mobile genetic elements, prophages can transfer resistance genes between different isolates through horizontal transfer. Therefore, continuous monitoring of *L. monocytogenes* resistance is still needed to assess the potential risk of human infections and guide public health interventions. In this study, the resistance breakpoints for TET, VAN, CIP were based on those of *Staphylococcus aureus*, which may have limitations, potentially leading to cases where resistance genes are present but the resistance phenotype is sensitive. In such cases, the combination of resistance phenotype and genotype is necessary.

## Conclusion

This study provides comprehensive insights into the prevalence and molecular characteristics of *L. monocytogenes* contamination in Chinese chilled pot skewer products in Beijing in 2019 and 2021. The high contamination rate (20.3%) across all product categories raises significant food safety concerns in this popular RTE food sector. The predominance of high-risk clones ST87 and ST121 suggests that these products may serve as potential vehicles for listeriosis transmission. The considerable genetic diversity revealed by cgMLST analysis indicates the presence of multiple contamination sources within the production environments. Moreover, the continuous detection of *L. monocytogenes* strains in specific manufacturers highlights possible shortcomings in sanitation protocols and facility hygiene management. Genomic similarities between foodborne and clinical isolates underscore the potential public health risks posed by contaminated chilled pot skewer products. However, this study has some limitations. No environmental samples were obtained, thus the tracing of contamination sources were restricted. Sample collection was restricted to specific regions and time periods, and no direct epidemiological data were available to establish casual links between food isolates and human cases. A coordinated One Health strategy is imperative in the future. This should include: (1) Enhanced environmental monitoring: regular and rigorous sampling of food production environments to identify and eliminate persistent contamination sources. (2) Integrated genomic surveillance: expanding WGS-based surveillance to routinely compare isolates from food, environment, and human patients to enable rapid source attribution and outbreak detection. (3) Consumer education: informing high-risk populations about the potential risks associated with consuming such RTE products without further treatment. By adopting this integrated approach, we can better protect public health against this formidable foodborne pathogen.

## Data Availability

The datasets presented in this study can be found in online repositories. The names of the repository/repositories and accession number(s) can be found below: https://nmdc.cn/resource/genomics/project/detail/NMDC10019942, NMDC10019942.

## Footnotes

^1^ https://github.com/MDU-PHL/LisSero

^2^ https://github.com/tseemann/mlst

^3^ https://nmdc.cn/resource/genomics/project/detail/NMDC10019942
