# Supplementary material for: Prevalence and characteristics of Listeria monocytogenes in ready-to-eat chilled pot skewer products
Source: Front Microbiol. 2025 Oct 6;16:1681344. doi: 10.3389/fmicb.2025.1681344 (PMC12536008; doi:10.3389/fmicb.2025.1681344)
Supplement: Supplementary file 1 [file Table_1.DOCX]

Supplementary Table1 Basic information of 340 chilled pot skewer samples in the study

| Manufacture | Number of Samples | | | Number of Positive Samples（percentage) | | | Number of Isolates |
| --- | --- | --- | --- | --- | --- | --- | --- |
|  | 2019 | 2021 | total | 2019 | 2021 | total |  |
| SS | 0 | 8 | 8 | 0 | 8 (100%) | 8 (100%) | 10 |
| F | 0 | 38 | 38 | 0 | 6 (15.79%) | 6 (15.79%) | 6 |
| R | 8 | 24 | 32 | 2(6.25%) | 4(12.5%) | 6(18.75%) | 6 |
| AB | 0 | 8 | 8 | 0 | 4 (50%) | 4 (50%) | 4 |
| TT | 0 | 16 | 16 | 0 | 4 (25%) | 4 (25%) | 4 |
| E | 10 | 59 | 69 | 8 (11.59%) | 27(39.13%) | 35 (50.72%) | 39 |
| D | 3 | 0 | 3 | 2 (66.67%) | 0 | 2 (66.67%) | 2 |
| PP | 0 | 8 | 8 | 0 | 2 (25%) | 2 (25%) | 2 |
| AF | 0 | 2 | 2 | 0 | 1 (50%) | 1 (50%) | 1 |
| BB | 2 | 0 | 2 | 1 (50%) | 0 | 1 (50%) | 1 |
| MM | 4 | 0 | 4 | 1 (25%) | 0 | 1 (25%) | 1 |
| A | 0 | 1 | 1 | 0 | 0 | 0 | 0 |
| AA | 1 | 0 | 1 | 0 | 0 | 0 | 0 |
| AC | 0 | 2 | 2 | 0 | 0 | 0 | 0 |
| AD | 0 | 4 | 4 | 0 | 0 | 0 | 0 |
| AE | 0 | 2 | 2 | 0 | 0 | 0 | 0 |
| AG | 0 | 2 | 2 | 0 | 0 | 0 | 0 |
| AH | 0 | 2 | 2 | 0 | 0 | 0 | 0 |
| B | 2 | 0 | 2 | 0 | 0 | 0 | 0 |
| C | 0 | 4 | 4 | 0 | 0 | 0 | 0 |
| CC | 2 | 0 | 2 | 0 | 0 | 0 | 0 |
| DD | 2 | 0 | 2 | 0 | 0 | 0 | 0 |
| EE | 2 | 0 | 2 | 0 | 0 | 0 | 0 |
| FF | 1 | 0 | 1 | 0 | 0 | 0 | 0 |
| G | 4 | 0 | 4 | 0 | 0 | 0 | 0 |
| GG | 2 | 0 | 2 | 0 | 0 | 0 | 0 |
| H | 4 | 6 | 10 | 0 | 0 | 0 | 0 |
| HH | 2 | 0 | 2 | 0 | 0 | 0 | 0 |
| I | 0 | 8 | 8 | 0 | 0 | 0 | 0 |
| II | 2 | 0 | 2 | 0 | 0 | 0 | 0 |
| J | 1 | 0 | 1 | 0 | 0 | 0 | 0 |
| JJ | 1 | 0 | 1 | 0 | 0 | 0 | 0 |
| K | 2 | 0 | 2 | 0 | 0 | 0 | 0 |
| KK | 2 | 0 | 2 | 0 | 0 | 0 | 0 |
| L | 2 | 0 | 2 | 0 | 0 | 0 | 0 |
| LL | 1 | 0 | 1 | 0 | 0 | 0 | 0 |
| M | 2 | 0 | 2 | 0 | 0 | 0 | 0 |
| N | 9 | 0 | 9 | 0 | 0 | 0 | 0 |
| NN | 1 | 0 | 1 | 0 | 0 | 0 | 0 |
| O | 2 | 6 | 8 | 0 | 0 | 0 | 0 |
| OO | 2 | 0 | 2 | 0 | 0 | 0 | 0 |
| P | 2 | 0 | 2 | 0 | 0 | 0 | 0 |
| Q | 4 | 0 | 4 | 0 | 0 | 0 | 0 |
| QQ | 0 | 2 | 2 | 0 | 0 | 0 | 0 |
| RR | 0 | 2 | 2 | 0 | 0 | 0 | 0 |
| S | 2 | 0 | 2 | 0 | 0 | 0 | 0 |
| T | 2 | 0 | 2 | 0 | 0 | 0 | 0 |
| U | 2 | 0 | 2 | 0 | 0 | 0 | 0 |
| UU | 0 | 2 | 2 | 0 | 0 | 0 | 0 |
| V | 2 | 0 | 2 | 0 | 0 | 0 | 0 |
| W | 2 | 0 | 2 | 0 | 0 | 0 | 0 |
| WW | 0 | 6 | 6 | 0 | 0 | 0 | 0 |
| X | 1 | 0 | 1 | 0 | 0 | 0 | 0 |
| XX | 0 | 8 | 8 | 0 | 0 | 0 | 0 |
| Y | 2 | 0 | 2 | 0 | 0 | 0 | 0 |
| YY | 0 | 6 | 6 | 0 | 0 | 0 | 0 |
| Z | 5 | 12 | 17 | 0 | 0 | 0 | 0 |

Supplementary Table2 Basic information of *L. monocytogenes* islated from chilled pot skewer samples in the study

| Isolate number | Sample types | Isolation time | Manufacture | Serogroup | ST | CC |
| --- | --- | --- | --- | --- | --- | --- |
| 438 | mixed skewer | 2019 | D | 1/2a,3a | 8 | 8 |
| 439 | mixed skewer | 2019 | R | 1/2a,3a | 101 | 101 |
| 440 | mixed skewer | 2019 | R | 1/2a,3a | 101 | 101 |
| 441 | mixed skewer | 2019 | E | 1/2b,3b,7 | 5 | 5 |
| 442 | mixed skewer | 2019 | E | 1/2b,3b,7 | 5 | 5 |
| 443 | mixed skewer | 2019 | E | 1/2b,3b,7 | 3 | 3 |
| 444 | mixed skewer | 2019 | E | 1/2b,3b,7 | 3 | 3 |
| 445 | mixed skewer | 2019 | E | 1/2b,3b,7 | 87 | 87 |
| 446 | mixed skewer | 2019 | E | 1/2a,3a | 121 | 121 |
| 447 | mixed skewer | 2019 | E | 1/2b,3b,7 | 5 | 5 |
| 465 | meat skewer | 2019 | D | 1/2a,3a | 121 | 121 |
| 473 | mixed skewer | 2019 | E | 1/2b,3b,7 | 5 | 5 |
| 474 | mixed skewer | 2019 | BB | 1/2a,3a | 8 | 8 |
| 494 | meat skewer | 2019 | MM | 1/2a,3a | 121 | 121 |
| 571 | meat skewer | 2021 | E | 1/2a,3a | 121 | 121 |
| 572 | vegetarian skewer | 2021 | E | 1/2c,3c | 9 | 9 |
| 573 | vegetarian skewer | 2021 | E | 1/2b,3b,7 | 3 | 3 |
| 574 | mixed skewer | 2021 | E | 1/2a,3a | 121 | 121 |
| 575 | mixed skewer | 2021 | E | 1/2a,3a | 121 | 121 |
| 576 | mixed skewer | 2021 | E | 1/2a,3a | 121 | 121 |
| 577 | mixed skewer | 2021 | E | 1/2a,3a | 121 | 121 |
| 578 | meat skewer | 2021 | R | 1/2b,3b,7 | 87 | 87 |
| 579 | vegetarian skewer | 2021 | SS | 1/2b,3b,7 | 87 | 87 |
| 580 | meat skewer | 2021 | SS | 1/2a,3a | 37 | 37 |
| 581 | mixed skewer | 2021 | R | 1/2c,3c | 1113 | 9 |
| 582 | vegetarian skewer | 2021 | E | 1/2b,3b,7 | 87 | 87 |
| 583 | mixed skewer | 2021 | E | 1/2a,3a | 121 | 121 |
| 591 | vegetarian skewer | 2021 | E | 1/2a,3a | 121 | 121 |
| 592 | vegetarian skewer | 2021 | PP | 1/2a,3a | 37 | 37 |
| 593 | meat skewer | 2021 | PP | 1/2b,3b,7 | 87 | 87 |
| 595 | vegetarian skewer | 2021 | AB | 1/2a,3a | 155 | 155 |
| 596 | mixed skewer | 2021 | AB | 1/2b,3b,7 | 87 | 87 |
| 611 | vegetarian skewer | 2021 | E | 1/2b,3b,7 | 87 | 87 |
| 612 | meat skewer | 2021 | E | 1/2b,3b,7 | 87 | 87 |
| 623 | meat skewer | 2021 | F | 1/2c,3c | 1113 | 9 |
| 624 | vegetarian skewer | 2021 | R | 1/2c,3c | 1113 | 9 |
| 625 | meat skewer | 2021 | R | 1/2c,3c | 1113 | 9 |
| 639 | mixed skewer | 2021 | TT | 1/2a,3a | 8 | 8 |
| 662 | vegetarian skewer | 2021 | SS | 1/2b,3b,7 | 87 | 87 |
| 663 | meat skewer | 2021 | SS | 1/2b,3b,7 | 87 | 87 |
| 664 | meat skewer | 2021 | E | 1/2b,3b,7 | 5 | 5 |
| 665 | vegetarian skewer | 2021 | E | 1/2b,3b,7 | 87 | 87 |
| 666 | vegetarian skewer | 2021 | E | 1/2a,3a | 121 | 121 |
| 667 | mixed skewer | 2021 | E | 1/2c,3c | 9 | 9 |
| 668 | vegetarian skewer | 2021 | E | 1/2a,3a | 121 | 121 |
| 669 | mixed skewer | 2021 | E | 1/2b,3b,7 | 87 | 87 |
| 670 | mixed skewer | 2021 | E | 1/2b,3b,7 | 87 | 87 |
| 671 | vegetarian skewer | 2021 | E | 1/2a,3a | 121 | 121 |
| 672 | meat skewer | 2021 | TT | 1/2b,3b,7 | 5 | 5 |
| 673 | meat skewer | 2021 | TT | 1/2a,3a | 8 | 8 |
| 674 | vegetarian skewer | 2021 | TT | 1/2c,3c | 9 | 9 |
| 675 | vegetarian skewer | 2021 | E | 1/2b,3b,7 | 3 | 3 |
| 676 | mixed skewer | 2021 | E | 1/2a,3a | 121 | 121 |
| 677 | mixed skewer | 2021 | E | 1/2b,3b,7 | 3 | 3 |
| 678 | mixed skewer | 2021 | E | 1/2b,3b,7 | 3297 | 87 |
| 679 | vegetarian skewer | 2021 | E | 1/2b,3b,7 | 87 | 87 |
| 680 | vegetarian skewer | 2021 | E | 1/2b,3b,7 | 3297 | 87 |
| 681 | meat skewer | 2021 | SS | 1/2b,3b,7 | 87 | 87 |
| 682 | meat skewer | 2021 | SS | 1/2a,3a | 121 | 121 |
| 683 | vegetarian skewer | 2021 | SS | 1/2b,3b,7 | 87 | 87 |
| 684 | vegetarian skewer | 2021 | SS | 1/2b,3b,7 | 87 | 87 |
| 685 | mixed skewer | 2021 | F | 1/2c,3c | 1113 | 9 |
| 693 | mixed skewer | 2021 | AF | 1/2c,3c | 9 | 9 |
| 694 | meat skewer | 2021 | F | 1/2c,3c | 1113 | 9 |
| 695 | vegetarian skewer | 2021 | F | 1/2c,3c | 1113 | 9 |
| 696 | vegetarian skewer | 2021 | E | 1/2a,3a | 121 | 121 |
| 697 | vegetarian skewer | 2021 | E | 1/2a,3a | 121 | 121 |
| 698 | meat skewer | 2021 | F | 1/2c,3c | 1113 | 9 |
| 699 | vegetarian skewer | 2021 | F | 1/2c,3c | 1113 | 9 |
| 700 | vegetarian skewer | 2021 | E | 1/2b,3b,7 | 5 | 5 |
| 712 | mixed skewer | 2021 | AB | 1/2b,3b,7 | 87 | 87 |
| 713 | mixed skewer | 2021 | AB | 1/2b,3b,7 | 87 | 87 |
| 714 | vegetarian skewer | 2021 | E | 1/2a,3a | 378 | 19 |
| 715 | vegetarian skewer | 2021 | E | 1/2b,3b,7 | 5 | 5 |
| 716 | vegetarian skewer | 2021 | SS | 1/2b,3b,7 | 716 | 87 |
| 717 | meat skewer | 2021 | SS | 1/2b,3b,7 | 717 | 87 |

Supplementary Table3 Differences in *L. monocytogenes* positive detection rates, serogroup distribution, and ST distribution among different sample types.

|  | Meat skewer samples | Vegetarian skewer samples | Mixed meat-and-vegetable skewer samples | χ^2^ | P |
| --- | --- | --- | --- | --- | --- |
| Sample size | 110 | 128 | 102 |  |  |
| Number of positive samples | 17 | 25 | 27 |  |  |
| Positive detection rate | 15.45% | 19.53% | 26.47% | 4.044 | 0.132 |
| Serogroup |  |  |  | 0.108 | 0.925 |
| 1/2a,3a | 6 | 9 | 12 |  |  |
| 1/2b,3b | 8 | 14 | 14 |  |  |
| 1/2c,3c | 4 | 5 | 4 |  |  |
| ST |  |  |  | 0.255 | 0.764 |
| ST87 | 5 | 8 | 6 |  |  |
| ST121 | 4 | 6 | 7 |  |  |
| ST1113 | 4 | 3 | 2 |  |  |
| ST5 | 2 | 2 | 4 |  |  |
| other STs | 3 | 9 | 11 |  |  |
